# White targeted chromatographic screening method of Molnupiravir and its metabolite with degradation kinetics characterization and in-silico toxicity

**DOI:** 10.1038/s41598-023-44756-6

**Published:** 2023-10-20

**Authors:** Sara I. Aboras, Ahmed A. Megahed, Fawzy El-Yazbi, Hadir M. Maher

**Affiliations:** 1 Pharmaceutical Analytical Chemistry Department, Faculty of Pharmacy, University of Alexandria, El-Mesallah, Alexandria, 21521 Egypt; 2 Al-Basra Health Unit, Alamriya Medical Area, Ministry of Health, Alexandria, Egypt

**Keywords:** Medical research, Chemistry

## Abstract

SARS-CoV-2 virus triggered a worldwide crisis, with world nations putting up massive efforts to halt its spread. Molnupiravir (MLN) was the first oral, direct-acting antiviral drug approved for nasopharyngeal SARS-CoV-2 infection with favorable safety and tolerability profile. This study aims at determination of MLN and N4-hydroxycytidine (NHC), its main degradation product and its main metabolite, using sensitive, simple, and green HPLC–DAD method. Moreover, under different stress conditions using NaOH, HCl, neutral, H_2_O_2_, dry heat and sun light, the method was applied for MLN assay along with kinetics degradation investigation. The linearity range for MLN and NHC were both 0.1–100 µg/mL with LOD and LOQ of 0.013 & 0.043 and 0.003 & 0.011 µg/mL, for MLN and NHC, respectively. MLN was found to be extremely vulnerable to alkali hydrolysis compared with acid and dry heat degradation. In contrast, MLN was stable under conditions of oxidative, neutral, and sunlight-induced deterioration. Acid and alkali-induced degradation followed pseudo first-order kinetics model. In addition, LC–MS-UV was used to suggest the mechanism of the stress-induced degradation route and to characterize the eluted degradation products. Toxicities of both MLN and its degradation products were evaluated using ProTox-II and they were found to be negligibly harmful. The proposed HPLC–DAD was effectively used for the analysis of MLN in commercial pharmaceutical formulations. The proposed method for MLN determination after greenness and whiteness appraisal was found to be superior compared to the reported methods for MLN analysis.

## Introduction

Since the outbreak of the SARS-CoV-2 virus in the first quarter of 2020, a worldwide crisis was triggered, with world nations putting up massive efforts to halt the spread of COVID-19. COVID-19 produces a severe respiratory syndrome which progresses to major disorders affecting several organs in the body involving kidneys, liver, and the neurological system. Drug companies have been racing to create new vaccines and antiviral compounds against the causal virus SARS-CoV-2 since the outbreak began; nevertheless, this has not been an easy task. Despite the recent availability of numerous worldwide authorized vaccines, inactivity against novel SARS-CoV-2 genotypes, concerns of insufficient protection for immune-compromised individuals, and a large magnitude of public resistance induced by people's suspicion made the vaccination effort difficult^1^.

Molnupiravir (MLN) is an oral broad-spectrum antiviral medication- originally developed to treat Alphavirus infections- used to treat COVID-19 disease caused by the nasopharyngeal severe acute respiratory syndrome infectious virus^1^.The U.S. Food and Drug Administration (FDA) issued an emergency use authorization for MLN in December 2021 for the treatment of mild-to-severe COVID-19 in adults of positive results of direct SARS-CoV-2 viral infections. Moreover, in November 2021, the European Medicines Agency (EMA) released a recommendation on the use of MLN in adults with an elevated risk for severe COVID-19. MLN is an oral ribonucleoside prodrug which is first hydrolyzed by the cellular carboxy esterase enzyme into a new isobutyryl ester prodrug, N4-hydroxycytidine (NHC) which is further phosphorylated to get NHC triphosphate. This phosphorylated analogue is the active antiviral form which inhibits RNA-dependent RNA polymerase (RdRp) with interference in the viral RNA replication^3–5^. MLN is known for its high tolerability which could be attributed to the lack of hepatic metabolism and the relatively short duration of action. Therapeutic treatment of COVID-19 infections with MLN for 5 days has not been linked to any raised levels of serum aminotransferase and hence no hepatotoxicity. According to EMA, only mild adverse effects could be encountered including GIT disorders with potential risk of embryofetal toxicity and mutagenicity^5,6^. It is also noteworthy to mention that since both MLN and NHC neither affect drug metabolizing enzymes nor drug transporters, no drug interactions have been reported^6–8^.

A review of the literature for the published analytical methods for MLN determination revealed few results, none of which studied the degradation kinetics of MLN. One method was concerned only with MLN assay in presence of its degradation products (DPs) using HPLC–DAD without studying the degradation kinetics or characterizing any of the DPs^9^. Also, a qualitative non-validated method focusing on the characterization of MLN DPs using LC–MS/MS^10^ was reported. Micellar HPLC–DAD and spectroscopic methods were also applied for MLN determination with another anti-viral drug, favipiravir^11^. Moreover, one HPTLC method was published for the assay of MLN, favipiravir and ritonavir in pure form and in pharmaceutical preparations^12^. Earlier, MLN was analysed along with its metabolite (NHC) in biological fluids using LC–MS/MS^13^.

Drug stability has an impact on the product's safety and effectiveness; degrading impurities may result in a loss of potency and production of potential negative consequences. In order to guarantee the quality and safety of pharmaceuticals, it is crucial to achieve their chemical and physical stability^14^. Therefore, all pharmaceuticals should undergo stress degradation tests including degradation kinetics, as well as identification and characterisation of the formed DPs, according to ICH (International Council for Harmonization) recommendations^15^. In market, shelf-life of MLN capsules was up to 36 months as per the Administration for Strategic Preparedness and Response (ASPR) and the Food and Drug Administration (FDA).^16^ However, the forced degradation study accelerates the production of DPs in a shorter period allowing for easier identification and characterisation. Degradation kinetics determine the shelf life of pharmaceuticals in various environments. It can help to forecast specific crucial aspects and critical factors that can alter the quality of drugs while being stored^17^.

As a result, our research framework aims to develop and validate a stability-indicating HPLC–DAD for MLN determination under a variety of stress conditions, including acid and alkali-induced hydrolysis, oxidation, photolysis, and thermal stress. The developed method was optimised for quantifying the intact drug MLN and its main DP (NMC); hence our work is superior in this respect to the previous reports^9,10^. Degradation kinetics were also studied for the first time which is an advantage over the reported HPLC–DAD method^9^. Moreover, the proposed method was found to be more sensitive for MLN determination compared with the previous one^9^ with LOD and LOQ of 0.013 & 0.043 µg/mL, for the former, while 0.05 & 0.1 µg/mL for the latter. Accordingly, this work enabled sensitive qualitative and quantitative determination of MLN and NHC which are essential points in deducing the level of stabilization of intact drugs. Even though Amara et al. have analysed MLN and its metabolite NHC using LC–MS/MS^13^, the proposed HPLC–DAD method was advantageous regarding cost, simplicity, and method greenness. Mass spectrometry was also used in this work to characterise probable DPs and the degradation pathways were proposed. The proposed DPs along with the main drug, MLN, were tested for their in-silico toxicity using Pro Tox-II program^18,19^. In addition, no analytical review of the reported methods analysing MLN along with their greenness appraisal was found in the literature, so a concise review was discussed. Finally, White Analytical Chemistry (WAC)^20^ was assessed as a promising extension of the of Green Analytical Chemistry (GAC)^21^ and the proposed method was found to be the best in whiteness assessment compared with the previous reports^9–13^.

## Results and discussion

To carry out a detailed kinetic degradation study of MLN, it is essential to have a stability-indicating assay method which can separate MLN along with its DPs. Moreover, it is of great value to determine both MLN and its main DP, NHC, using the same method which implies the possibility of their determination in actual plasma samples and any future clinical studies.

The degradation products detected using LC–MS-UV, should be structurally elucidated to characterize its *in-silico* toxicity along with MLN. Therefore, Pro Tox- II was applied to assess their possible toxicity. Finally, the degree of greenness and whiteness of the proposed HPLC–DAD method was appraised and compared with the previously reported ones.

The potential role of DAD in assessing spectral peak purity is extremely beneficial to ensure peaks separation in stability-indicating assays^22^.

### Optimization of chromatographic conditions

Two reversed phase columns were tried namely Agilent HC-C18 (150 × 4.6 mm, 5-µm) and Agilent HC-C8 (150 × 4.6 mm, 5-µm). They were evaluated using mobile phases of ACN and deionized water in the ratio of 25:75, v/v, respectively. Chromatographic conditions were preliminary optimized to achieve good separation between MLN and NHC peaks. Experimental trials revealed that the two columns didn’t differ significantly in terms of the retention behavior of MLN and NHC. This could be attributed to the high hydrophilicity of MLN, log D = 0.46^23^. On the other hand, the peak area, and tailing factor of MLN and NHC were better using the C18 column compared with the C8 one.

Trying different pH values (3, 5 & 7) of the aqueous phase resulted in an insignificant effect on R_t_, peak area, or tailing factor of both compounds, owing to MLN pKa values (2.2, 10.2 & 12.0) which are 2 units away from the studied pH values^22^. Therefore, simply deionized water was selected for the study as it is better than buffer for the instrument pressure and hence maintaining the column lifetime. Both MeOH and ACN were tried as organic modifiers. Sharper and more symmetrical peaks of MLN and NHC were obtained with ACN compared with MeOH, T_f_ of 1.07 & 1.11 for the former compared with 1.3 & 1.24 for the latter, Supplementary Fig. 1. So mobile phases with ACN were used.

**Figure 1:**
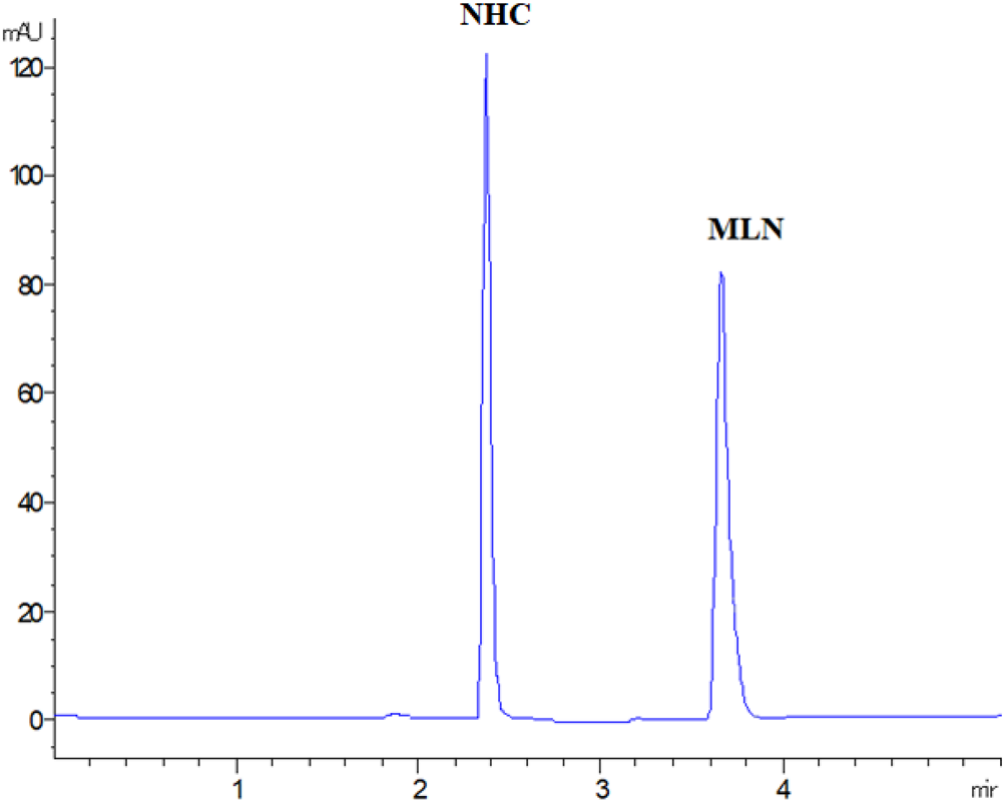
HPLC DAD chromatogram of 10 µg/mL MLN and NHC using the proposed method.

**Figure 2:**
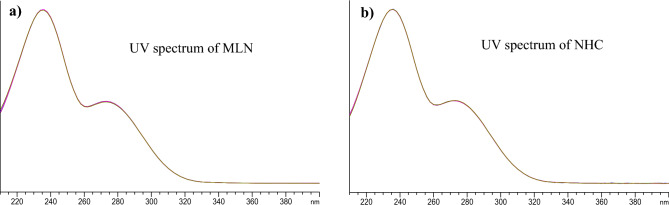
Purity profile of the UV spectrum of MLN, **a**), and NHC, **b**).

In this work, isocratic elution was satisfying when developing the stability-indicating method in presence of stress degradation conditions with Rs > 2 between MLN and NHC, being the main DP and the closest peak to the intact drug. This was confirmed in our method by monitoring the purity plots of MLN and NHC peaks under different stress conditions. Purity factors within the corresponding automatically computed noise threshold limits indicated pure peaks. As a result, the following chromatographic conditions were applied, Agilent HC-C18 analytical column (150 × 4.6 mm, 5 µm) with a mobile phase system consisting of solvent A (deionized water) and solvent B (ACN) in an isocratic elution 75:25, v/v, respectively. The run time was 5 min with retention times of 2.9 and 3.7 min for NHC and MLN, respectively, and the detection wavelength was 236 nm corresponding to the λ_max_ for MLN and NHC, Fig. 1. UV spectra of both MLN and NHC, indicating similar spectra and similar λ_max_,_._ were shown in Fig. 2. System suitability parameters for the determination of MLN with NHC indicated that the two compounds were eluted within reasonable time with high number of theoretical plates indicating high column efficiency, and with good resolution (Rs more than 2), Table 1. The obtained results were within the acceptance criteria according to FDA^24^, indicating an acceptable degree of system suitability.

**Table 1:** System suitability parameters for the HPLC–DAD determination of MLN and NHC^a^.

Parameters	MLN	NHC
t_R_ ± SD (min)^b^	3.7 min ± 0.01	2.4 min ± 0.01
Capacity factor (k')	3.83	3.19
Selectivity (α)	1.2	
Theoretical plates (N)	13,467	10,085
Resolution (R_s_)^c^	7.5	
Tailing factor (T_f_)	1.07	1.11

^a^System suitability recommendations: k' (1–10), N > 2000, α > 1, R_s_ > 2 and T_f_ (0.8–1.2)^20^.

^b^Average t_R_ ± SD of three determinations.

^c^Resolution between the drug and the DP1.

### Forced degradation and stability-indicating study

As per ICH recommendations^15^, MLN was exposed to various stress conditions including acid, base hydrolysis, neutral, oxidative, dry heat, and photo-degradation. Degradation behavior of MLN was monitored by the proposed HPLC–DAD method.

The same stability study results were obtained for assaying the drug either as a standard solution or as a tablet extract. Different stress conditions, Table 2, were preliminary tested. Initially, relatively mild conditions resulted in minor degradation (ex: 10%H_2_O_2_, 1 M HCl/ 80 °C) except for NaOH. Alkaline degradation was tested using NaOH solution of different strengths at room temperature (1 M, 0.1 M, 0.01 M). It was found that both 1 M and 0.1 M NaOH resulted in complete degradation at room temperature (RT) within 15 min. Thus, degradation kinetics was investigated using milder alkalinity (0.01 M NaOH) at room temperature. After exposure to the different stress conditions, %recovery of MLN was calculated at different time intervals. It was found that MLN is very susceptible to alkaline hydrolysis and to a less extent to acidic hydrolysis. This was confirmed by the decrease in the peak area of MLN (R_t_ = 3.7 min ± 0.05) to 17.8% with 0.01 M NaOH at RT for 2.5 h compared with 30% with 2 M HCl at 60°C for 30 min. These degradation conditions were accompanied by the appearance of a DP1 peak, NHC, at Rt (2.4 min ± 0.06) with a UV spectrum similar to MLN, Fig. 2. Spiking with standard NHC confirmed the identity of the eluted DP1 peak to be NHC with the peak purity assessed by DAD. MLN degradation was attributed to the acidic or alkaline-induced hydrolysis of the ester group in MLN molecule. In both acidic and alkaline hydrolysis, protonation of the –COOH group was the first step in degradation. However, RCOO^−^ is isolated as sodium salt in alkaline hydrolysis and thus it is irreversible; additionally, the product RCOO^-^ stabilizes itself by resonance^25^. This explained the excessive alkaline degradation rather than the reversible acidic hydrolysis.

**Table 2:** Summary of the degradation behavior of MLN using the proposed HPLC–DAD method.

	Degradation conditions	% Recovery
Alkaline degradation	0.01 M NaOH, RT, 2.5 h	18%
Acidic degradation	2 M HCl, 60°C, 30 min	30%
Oxidative degradation	30% H_2_O_2_, 60°C, 2h	101%
Neutral degradation	H_2_O, 80 °C, 30 min	99%
Dry heat degradation	Dry heat 100°C, 2h	85%
Photolytic degradation	Sun light, 4h (summer)	98%

DP1 is the free alcohol of MLN and is known as ß-d-N4-hydroxycytidine (NHC), the primary active metabolite of MLN which is usually monitored in plasma when studying the pharmacokinetics of MLN^26,27^. Our proposed method was able to separate NHC from MLN with high purity as supported with DAD and thus it could be applied for further bioanalytical studies. This conclusion was verified using LC–MS–UV as discussed later.

**Figure 3:**
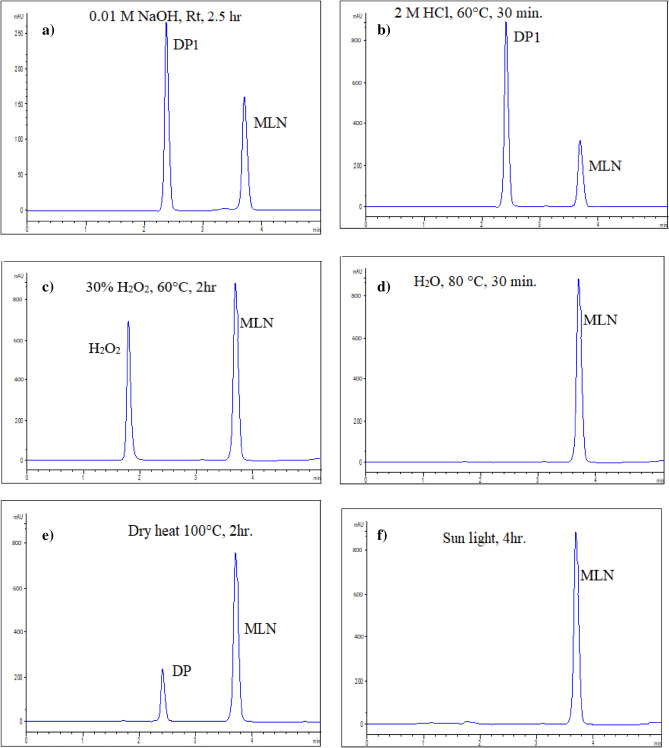
HPLC–DAD chromatograms of 100 µg/mL of MLN after alkali-induced degradation (0.01 M NaOH, RT, 2.5 h) showing 1 DP, (**a**), acid-induced degradation (2 M HCl, 60°C, 30 min.) showing 1 DP (**b**), oxidative-induced degradation (30% H_2_O_2_, 60°C, 2 h) showing no DP, (**c**), water-induced degradation (H_2_O, 80 °C, 30 min) showing no DP, (**d**), dry heat-induced degradation (100°C, 2 h) showing 1 DP, (**e**) photo-degradation (day light, 4 h) showing no DP of MLN, (**f**).

**Figure 4:**
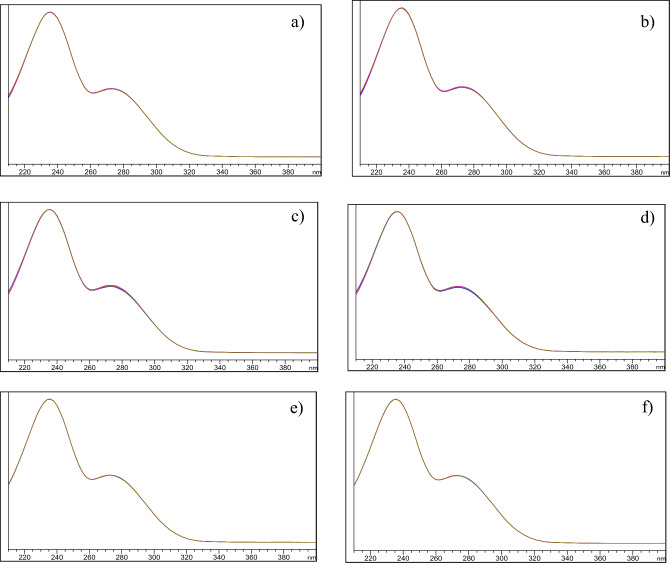
Purity profiles of MLN peaks obtained from HPLC–DAD for alkaline, (**a**), acidic, (**b**), oxidative, (**c**) neutral, (**d**), dry heat, (**e**), and sunlight, (**f**), degradations.

**Figure 5:**
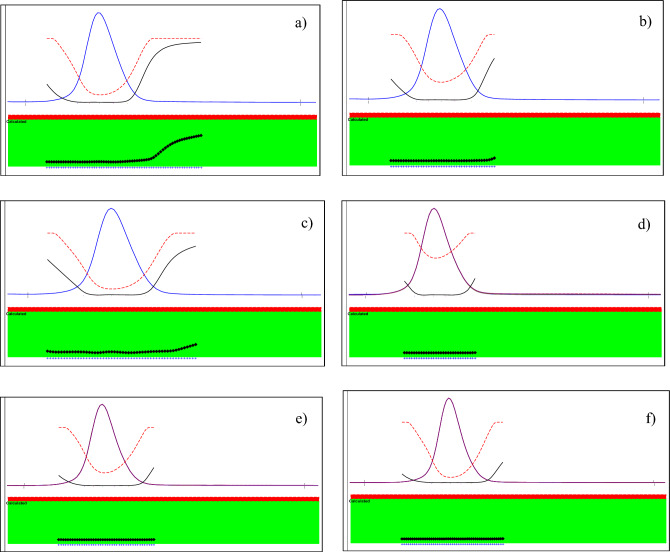
Purity plots of MLN peaks obtained from HPLC for alkaline, (**a**), acidic, (**b**) oxidative, (**c**), neutral, (**d**) dry heat, (**e**), and sunlight, (**f**), degradations.

On the other hand, MLN exhibited relatively higher stability under the studied oxidative, photolytic (direct sun light for 4 h), and neutral stress conditions (80 °C for 30 min), since no additional peaks other than the intact MLN peak appeared in its respective chromatogram with retained recovery of 100% ± 2%, Table 2. Using dry heat conditions in oven at 100 °C for 2 h, the % recovery of MLN declined to 85%, with DP eluted at Rt (2.9 min ± 0.06), corresponding to NHC. The chromatograms of MLN under different stress conditions were shown in Fig. 3. After being exposed to different stress conditions, % recovery of MLN was calculated, and the degradation behavior was in accordance with the previously published method^9^. The chromatographic peak purity was tested using DAD in all the previously stated stress degradation investigations. The absorption spectrum of MLN was acquired at different time intervals for all the specified stress investigations, and these spectra were superimposed to establish the purity of the peaks, Fig. 4. Peak purity was also evaluated using a peak purity plot. The purity factor of MLN peak was under the estimated threshold limit, confirming the purity of the MLN peak for all the mentioned stress experiments at various time intervals, Fig. 5.

### Validation of the proposed HPLC–DAD method

Analytical method validation of MLN and NHC was appraised for linearity, accuracy, precision, specificity, and robustness in agreement with ICH guidelines^15^.

### Linearity and concentrations ranges

A series of different concentrations of MLN and NHC were analyzed to evaluate the linearity of the proposed HPLC–DAD method. Under the optimized conditions, the method was linear in the concentration range 0.1 to 100 µg/mL for both compounds. The computed linear regression equations for MLN and NHC were Y = −1.1 + 50.75X and Y = −4.5 + 37.8X, respectively. The high correlation coefficient (r = 0.9996 for MLN and 0.9999 for NHC, r^2^ = 0.9992 for MLN and 0.9998 for NHC) combined with low intercepts demonstrated good linearity. Furthermore, the RSD % of the slope (Sb %) was evaluated and was found to be 0.7 for MLN and 0.05 for NHC (less than 2%) indicating that a minor deviation around the slope was produced^28,29^. Furthermore, the small significance F values, 2.23 × 10^–7^ and 3.46 × 10^–11^ for MLN and NHC, respectively, suggested that the scattering of the experimental data sets around the regression line was minimal.

### Detection and quantitation limits

Limits of detection (LOD) and of quantitation (LOQ) were derived from MLN & NHC concentrations with a signal–to–noise ratio of 3:1 for LOD and 10:1 for LOQ. The high sensitivity of the proposed HPLC method was confirmed by the low LOD and LOQ of MLN and NHC, 0.013 & 0.043 and 0.003 & 0.011µg/mL, respectively.

### Accuracy and precision

Three concentrations of MLN and NHC (0.2, 2 & 80 µg/mL) were analyzed three times on the same day for intra-day and on three consecutive days for inter-day validation. Relative error (E_r_) and relative standard deviations (RSD) were calculated to evaluate the method’s accuracy and precision, respectively, Table 3. The method's accuracy and precision were demonstrated by the low values (less than 2%) of both E_r_ and RSD percentage, respectively in addition to excellent recovery calculations (98–102%) using the developed linear regression Eq. ^28^.

**Table 3:** Intra-day and inter-day accuracy and precision for the determination of MLN and NHC.

Concentration µg/mL	MLN			NHC		
	% Recovery ± SD^a^	RSD (%)^b^	E_r_ (%)^c^	% Recovery ± SD^a^	RSD (%)^b^	E_r_ (%)^c^
Intra-day accuracy and precision						
0.2	99.90 ± 1.74	1.74	−0.10	100.14 ± 1.91	1.90	0.14
2	99.62 ± 1.25	1.24	−0.38	99.62 ± 1.69	1.70	−0.38
80	99.77 ± 0.67	0.67	−0.23	101.99 ± 0.27	0.27	1.99
Inter-day accuracy and precision						
0.2	99.16 ± 1.92	1.90	−0.84	99.88 ± 1.54	1.53	−0.12
2	100.50 ± 0.49	0.49	0.50	100.27 ± 1.69	1.68	0.27
80	101.13 ± 0.26	0.27	1.13	101.37 ± 1.53	1.51	1.37

^a^Mean ± standard deviation for three determinations.

^b^% Relative standard deviation.

^c^% Relative error.

### Specificity

The specificity was appraised by the absence of interfering substances from regularly found excipients and inactive ingredients in the dosage form, as indicated later in the section “Assay of pharmaceutical formulation”. Furthermore, the method was able to separate MLN from NHC with a satisfactory resolution (greater than 2) for successful quantification, according to FDA guidelines^24^. Furthermore, peak purity was determined by DAD, which assured MLN peak purity during its analysis with the presence of excipients (tablets analysis) or probable degradants (stress degradation study).

### Robustness

Column chromatographic conditions were changed slightly to verify the method robustness, including the working wavelength (± 1 nm), mobile phase ratio (± 2%), water pH (± 0.2 pH units), and flow rate (± 0.1 mL/min). There was no discernible effect on the measured responses or retention times when these modifications were applied. MLN and NHC peak area RSD% did not exceed 2% under these modifications. In addition, MLN and NHC peak' retention time had minimal SD values, Table 4.

**Table 4:** Evaluation of the robustness of the proposed HPLC method for the determination of MLN and NHC.

Parameters	MLN			NHC		
	Mean % recovery ± SD ^a^	RSD% ^b^	t_R_ ± SD ^c^	Mean % recovery ± SD ^a^	RSD% ^b^	t_R_ ± SD ^c^
Ratio of organic modifier mobile phase (25% ± 2%)	100.03 ± 0.06	0.06	3.70 ± 0.30	101.07 ± 0.40	0.40	2.40 ± 0.42
Wavelength of determination (236 ± 1 nm)	99.64 ± 0.03	0.03		99.81 ± 0.29	0.30	
pH of water (7 ± 0.2 pH units)	100.11 ± 0.07	0.07	3.70 ± 0.01	100.94 ± 1.22	1.22	2.40 ± 0.10
Flow rate (± 0.1 mL/min)	99.31 ± 0.15	0.15	3.70 ± 0.82	100.56 ± 0.98	0.97	2.40 ± 1.01

^a^Mean of percentage recovery of peak area of MLN & NHC of concentration 10 µg/mL at the studied parameters ± SD.

^b^Percentage relative standard deviation of % recovery of MLN & NHC at the studied parameters.

^c^Mean of retention time of MLN at the studied parameters ± SD.

### Stability of solutions

For MLN and NHC calibration standards and sample solutions, no chromatographic changes were noticed within 6 h at RT. Also, for one week, the stock solutions, prepared in HPLC-grade MeOH, were found to be stable when kept in the freezer at 0 °C. This was confirmed by the absence of any additional peaks besides unchanged retention times and peak areas of the MLN and NHC.

### Assay of pharmaceutical formulations

The proposed HPLC–DAD method was used for the determination of MLN in its dosage form (Molnupiravir-Eva Pharma®). MLN was eluted at its retention time with no interfering peaks observed from any of the excipients, Supplementary Fig. 2a, Table 5. DAD permitted the peak purity verification as no interfering components were detected co-eluting with MLN peak. Furthermore, the validity of our method was successfully assured by spiking the solution of dosage form (50 µg/mL) with the pure standard (standard addition method), Supplementary Fig. 2b, Table 5.

**Table 5:** Determination of MLN in Molnupiravir-Eva Pharma® capsules and spiked dosage form samples by the proposed HPLC–DAD method.

Pharmaceutical formulation		HPLC–DAD			
Molnupiravir-Eva Pharma® capsules (each pill containing 200 mg MLN)	%Found ± SD^a^	Spiking with the pure standard			
		Claimed (μg/ mL)	standard added (μg/ mL)	standard found (μg/ mL)	%Recovery of the standard added^b^ (μg/ mL)
	MLN 100.05 ± 0.23	50	2 10 20 Mean ± SD	2.03 9.96 20.41	101.5 99.6 102.0 101.03 ± 1.25

^a^Average of five determinations.

^b^Average of three determinations.

### Investigation of degradation kinetics

**Figure 6:**
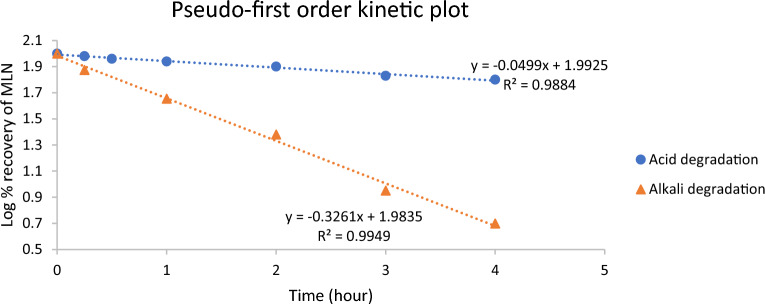
Pseudo first-order kinetic plots for 100.0 μg/mL MLN under acid (2M HCl) and alkaline (0.01 M NaOH) stress conditions at room temperature.

Following the development of the new anti-COVID-19 drug, MLN, a need to investigate its kinetic behavior was recalled. This was achieved by estimating the remaining MLN concentration (C_t_) after being degraded at different time (t) intervals under acidic (2M HCl) and alkaline (0.01 M NaOH) degradation conditions, at RT. Kinetics of acid and alkali hydrolysis were studied through estimation of MLN degradation rate constant (K) and half-life (t_1/2_) at RT at the mentioned conditions to estimate the on-shelf stability of MLN. When the degradation procedures in both HCl and NaOH were induced and monitored using the proposed HPLC–DAD method, a regular decline in MLN concentration was observed as the time intervals increased. Through plotting log MLN remaining concentration (C_t_) versus time in both acidic and alkaline conditions, a linear relationship was obtained, Fig. 6, indicating pseudo first-order degradation kinetics. According to the following equation^17^:

$$\mathrm{log}\left(Ct\right)=\mathrm{log}\left(Co\right)- \frac{kt}{2.303}$$

$${t}_\frac{1}{2}=\frac{0.693}{{k}_{obs}}$$

where, C_t_ is MLN remaining concentration, after time (t).

C_o_ is the initial concentration of MLN,

K, with negative sign, is the apparent first order rate constant.

For MLN acid and alkali hydrolysis, the half-life time (t_1/2_) calculated from Fig. 6 were 6.58 and 0.88 h, respectively with corresponding observed reaction rate constants (k_obs_) of 0.11 h^−1^ and 0.79 h^−1^, respectively.

### LC–MS-UV study of MLN and its degradation products

**Figure 7:**
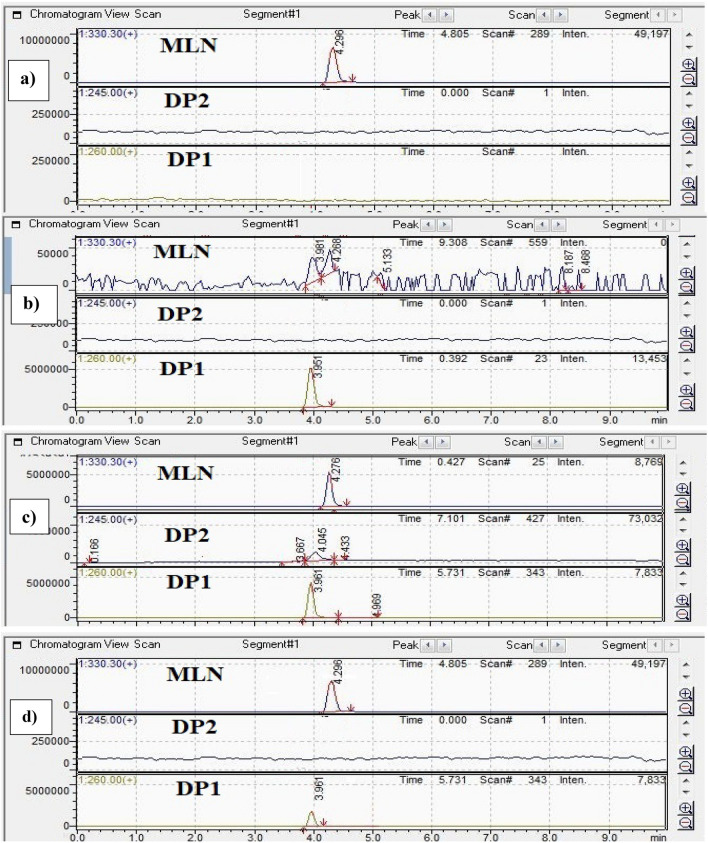
LC–MS-UV chromatograms of MLN standard, (**a**) and using different stress conditions: alkaline degradation, (**b**) acidic degradation, (**c**) and dry heat degradation, (**d**) with m/z 330, 260 and 245 for standard MLN, DP1 and DP2, respectively.

**Figure 8:**
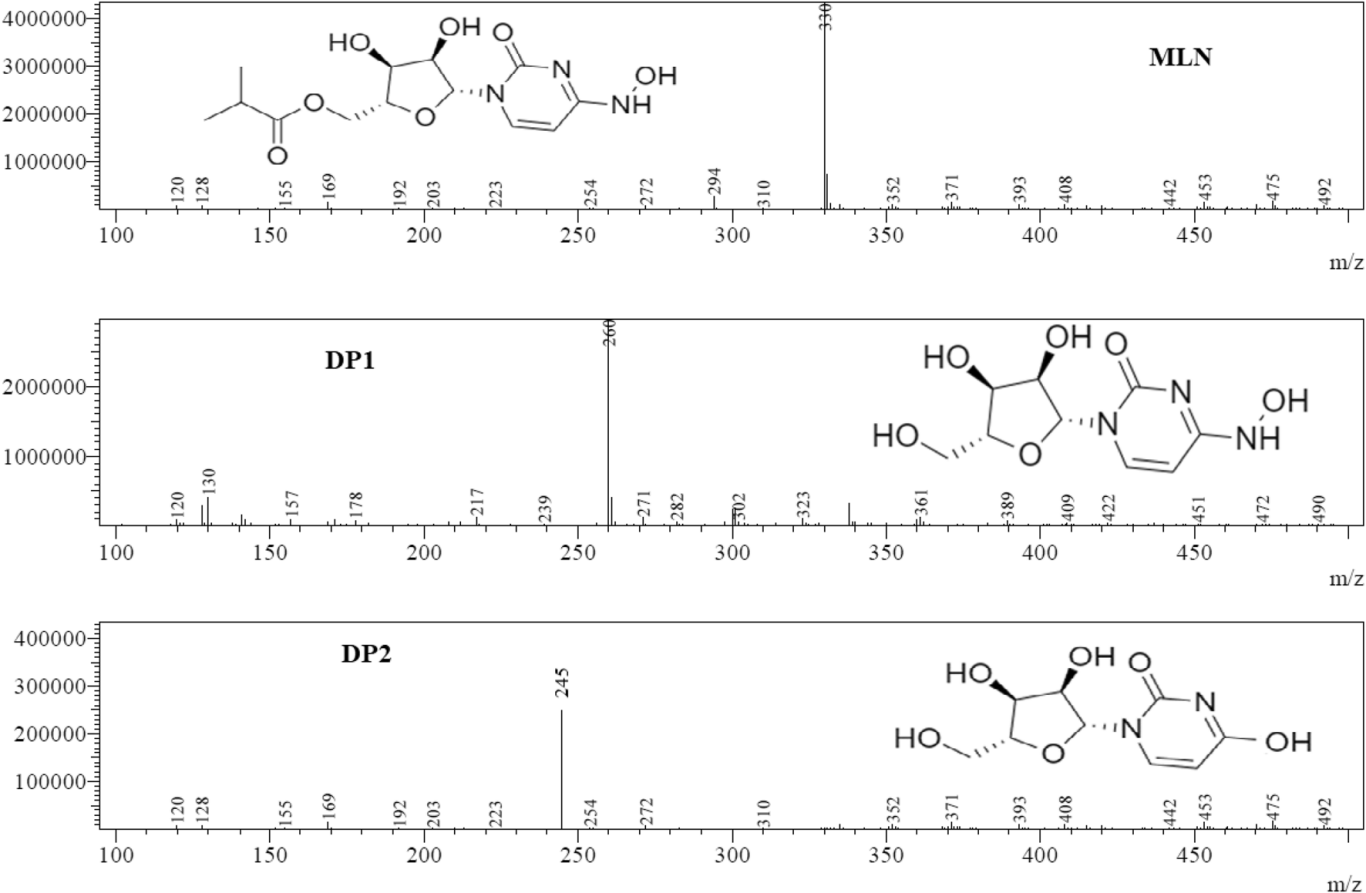
Mass spectrum of MLN and its proposed DPs with suggested corresponding chemical structures.

To elucidate and characterize the degradation pathway of MLN under acidic, alkaline, and dry heat conditions, degraded solutions were injected on an LC–MS–UV instrument and the m/z of the eluted peaks were recorded. DPs generated under acidic, alkaline, and dry heat conditions were separated and investigated using the optimized chromatographic method, Fig. 7. The corresponding m/z values were recorded through the scan mode giving a fragment with m/z = 260 for all the three stress conditions, besides the fragment with m/z = 245 which appears only in acidic degradation conditions, Fig. 8.

**Figure 9:**
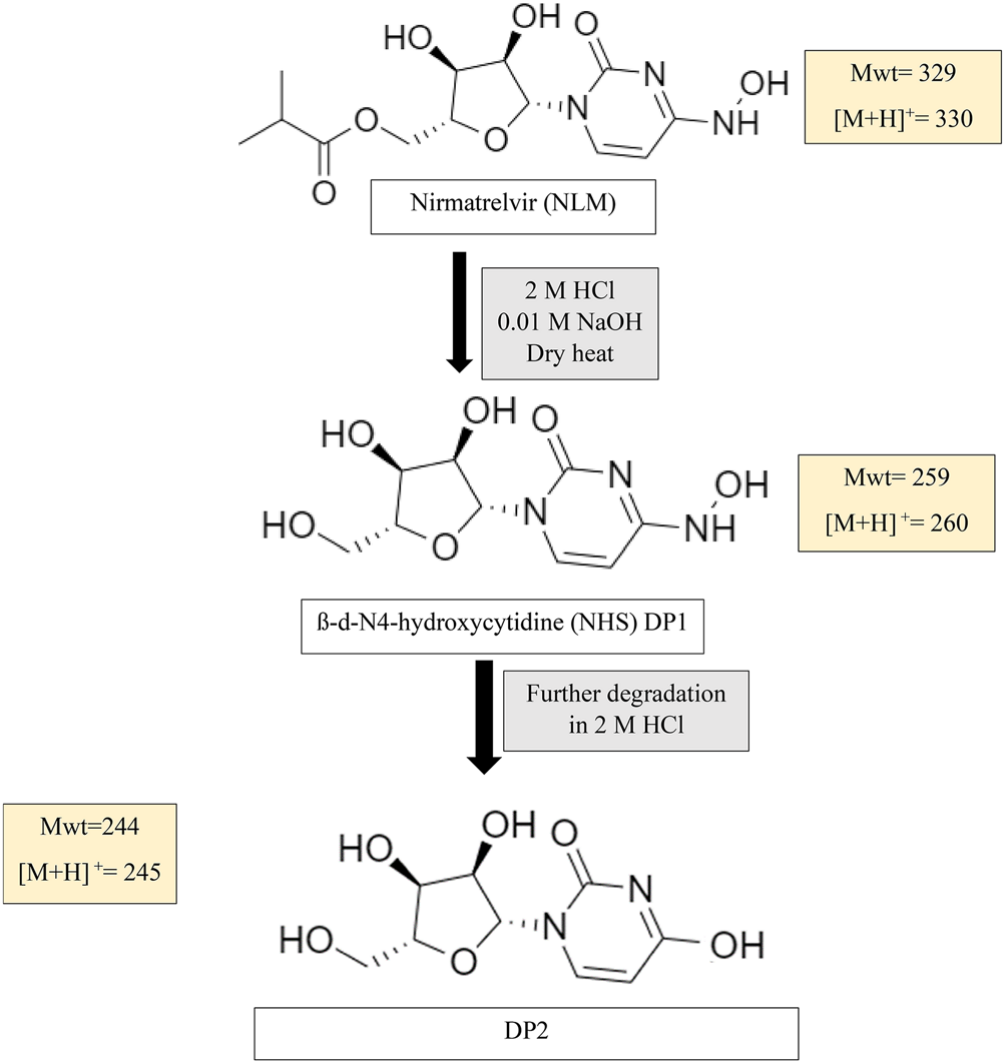
The proposed degradation pathway of MLN in presence of HCL, NaOH and dry heat degradation conditions.

The assumed pathway was shown in Fig. 9 and it was in accordance with the previously published method regarding acidic degradation^10^. The alkaline degradation in our proposed method yielded DP1, NHC, with m/z = 260. It is noteworthy to mention that dry heat degradation wasn’t studied in the previous method^10^. Thus, our proposed method was the first to study the pathway of dry heat-induced degradation of MLN. It was assumed that the degradation starts with the cleavage of the ester group under acid, base, and dry heat conditions giving rise to the free alcohol (ß-d-N4-hydroxycytidine: NHC, Mwt = 259, m/z of [M + H] ^+^  = 260) and the corresponding acid 2-butanoic acid. NHC undergoes further decomposition through deamination and hydroxylation of the pyrimidine ring in the acidic degradation condition, to form DP2, m/z of [M + H] ^+^  = 245^10^. DP2 did not appear in the proposed HPLC–DAD method may be due to its low yield and lower sensitivity of DAD compared with the MS detector^30–32^.

### In-silico toxicity studies

The widely used computational toxicity web server, ProTox-II, is used to anticipate several toxicity end points. It includes molecular and chemical target information, such as molecular similarity, pharmacophores, fragment tolerance, and computer models. ProTox-II models are validated using probability-based CLUSTER cross-validation with selective oversampling for organ toxicity, toxicity endpoints (carcinogenicity, mutagenicity, cytotoxicity), and toxicological pathways^33^.

Different toxicity potentials of MLN and its DPs were forecasted using this accessible database. Hepatotoxicity, mutagenicity, immunotoxicity, carcinogenicity, and cytotoxicity were involved in the evaluation. For MLN and the two DPs, the toxicity endpoint as well as organ toxicity were estimated. According to Table 6, MLN has been demonstrated to exhibit hepatotoxicity with a 0.56 (56%) confidence level. On the other hand, with a confidence level of 0.66 (66%), 0.59 (59%) and 0.60 (66%), DP1 was predicted to be hepatotoxic, carcinogenic, and mutagenic, respectively. DP2 showed hepatotoxicity, carcinogenicity, and mutagenicity with confidence levels of 0.63 (63%), 0.55 (55%), and 0.58 (58%), respectively. As the confidence levels of organ toxicity and toxicity end points were below 0.7, this indicated that MLN and its degradation products were below threshold^33^. Therefore, there is no high evidence for their organ toxicity and toxicity end points.

**Table 6:** Predicted probability of toxicity profile of Molnupiravir and its degradation products (DP1: NHC & DP2).

Classification	Target	Shorthand	Drug	DP 1	DP 2
LD 50 (mg/kg)	–	–	826	826	1640
Toxicity Class	–	–	4	4	4
Hepatotoxicity	Hepatotoxicity	dili	0.56	0.66	0.63
Carcinogenicity	Carcinogenicity	carcino	NA	0.59	0.55
Immunotoxicity	Immunotoxicity	immuno	NA	NA	NA
Mutagenicity	Mutagenicity	mutagen	NA	0.60	0.58
Cytotoxicity	Cytotoxicity	cyto	NA	NA	NA

Furthermore, acute toxicity of MLN and its DPs was evaluated through their ability to kill half of the test animals (rodents) within 24 h of exposure (LD50)^33^. LD50 of MLN, DP1, and DP2 were 826, 826, and 1640 mg/kg, respectively. LD50 values are divided into six classes with class I being the most lethal and class VI being the least poisonous^33^. Various toxicity endpoints for MLN and DPs were anticipated using computerized studies. According to a worldwide harmonized approach called "classification and labelling of chemicals" based on LD50 (mg/kg) values from Protox-II, MLN and its DPs were in toxicity class IV, meaning that they are harmful when swallowed^33^.

Even though the reported LC–MS/MS^10^ method for degradants characterization used the same software for in-silico toxicity assessment of MLN and its DPs, ProTox-II, the authors did not demonstrate the predicted probability of each toxicity end point which is a vital key for either neglecting or accepting the suggested toxicity. Thus, this study is advantageous in this aspect.

### Greenness of the method

In our study, we have applied three tools of greenness assessment: the Analytical Eco-Scale^34^, the green analytical procedure index (GAPI)^35^ and the novel Analytical Greenness metric (AGREE)^36^ were applied for both assessment and comparison of greenness of our proposed stability indicating HPLC method with the other published chromatographic ones^9,11,13^. It is worthy to mention that all these previous methods focused only on the determination of MLN in presence of its degradants^9^, with its metabolite^13^, or with other anti-COVID-19 drugs^11,12^ without studying the degradation kinetics of MLN or even quantitating its main DP and metabolite, NHC. The Analytical Eco-Scale tool demonstrates the method’s greenness degree by a numerical value, its calculation relies on allotment of penalty points (PPs) for each category of the method that contradicts the greenness concept^34^. The evaluated categories include the hazardous reagents used, consumed power energy and any created waste volume, followed by subduction of the total calculated penalty points from 100. The method is then considered acceptable green if the resulting value is above 50 and of excellent greenness if its value is above 75. According to this tool, the calculated value of the proposed method was found to be more than 75 (Eco-Scale score = 84) indicating excellent greenness with the least possible negative effect on the environment, Table 7.

**Table 7:** The penalty points of the proposed chromatographic method according to the Analytical Eco-scale.

Reagents/Instruments	Penalty points (PPs)
Acetonitrile	3
NaOH	2
HCl	4
H_2_O_2_	4
Energy of HPLC	0
Occupational hazard	0
waste	3
PPs	16
Eco-scale score	84

In addition, two more greenness appraisal tools were applied for greenness comparison between the proposed method and the other chromatographic reported methods namely, the GAPI and AGREE tools. The GAPI tool allows for a more thorough evaluation of each analytical method's ecosystems. This tool evaluates the entire analytical technique, beginning with sample collection and preparation and ending with the final determination. It represents the method's eco-friendliness through a colorful pictograph with 15 elements that refer to 15 evaluated criteria of the process, including sample preparation and extraction, solvent and reagent characteristics, energy consumption, waste quantities, and occupational risks. Each portion is colored either green (for the greenest element) or yellow (for the least green element), with non-green characters receiving a red tint. Analytical methods for MLN determination were sorted using this tool based on a visual evaluation of the number of green and red zones in the GAPI pictographs, Table 8. The greenest method is the one with the largest number of green zones and the fewest number of red zones, thus the reported micellar HPLC–DAD and spectroscopic methods^11^ are the greenest (9 green, 5 yellow, and 1 red zones), because they use green and biobased mobile phase and solvent components (0.1 M SDS, 0.01 M Brij-35, and 0.02 M KH_2_PO_4_) and 0.1 M HCl, respectively. After that, the other HPLC–DAD methods, the reported^9^ and the proposed ones came in second place in greenness with 7 green, 6 yellow and 2 red zones, due to the use of less green mobile phase containing acetonitrile. The reported bioanalytical LC–MS/MS^13^ method came last having several sample handling and preparation steps, accompanied by the high energy consumption of the tandem mass detector.

**Table 8:** Summary of the chromatographic conditions of different method of MLN analysis with corresponding sensitivity, aim of work and AGREE and GAPI greenness assessment tools.

Method	Chromatographic conditions	Sensitivity μg/mL		Aim of the method	AGREE assessment	GAPI assessment
Proposed stability indicating HPLC–DAD	**- SP**: HC-C8 column (4.6 × 150 mm, 5 μm particle size) - **MP**: ACN: water (25:75 v/v) - RT: 5 min - FR: 1.0 mL/min	**Range:** **LOD:** **LOQ:**	0.1–100 0.013 0.043	-Stability indicating method -Degradation kinetics followed by structural elucidation		
Reported stability indicating HPLC–DAD^9^	- **SP:** HS C18 Column (75 × 4.6 mm, 3 μm particle size) - **MP**: ACN: water (20:80 v/v) - RT: 5 min - FR: 0.5 mL/min	**Range:** **LOD:** **LOQ:** **Tf:**	0.1–60 0.05 0.1 1.24	-Stability indicating method -Permeability quantitation in nanoformulations		
Reported Micellar HPLC–DAD^11^	**- SP:** -C18 core–shell column (5 µm, 150 × 4.6 mm) - **MP:** 0.1 M SDS, 0.01 M Brij-35, and 0.02 M KH_2_PO_4_ mixture and adjusted to pH 3.1 - RT: 5 min - FR: 1 mL/min	**Range:** **LOD:** **LOQ:**	0.5–50 0.02 0.05	-Analysis of MLN with Favipiravir -Study the dissolution behaviour if coformulated with Favipiravir		
Reported spectrophotometric method^11^	**- Dissolving the drug in 0.1 M HCl** **- 210–350 nm**	**Range:** **LOD:** **LOQ:**	6–22 Not mentioned	-Analysis of MLN with Favipiravir -Study the dissolution behaviour if coformulated with Favipiravir		
Reported Biological HPLC–MS/MS method^13^	**- SP:** polar Atlantis C18 column (2.1 × 100 mm, 3 μm) - **MP:** gradient elution of 1mM Ammonium acetate (pH4.3) in water (pH4.3) and 1mM Ammonium acetate in ACN - RT: 6 min - FR: 0.35 mL/min	**Range:** **LLOQ:**	2.5–5000 ng/ml 2.5 ng/ml	-Bioanalysis of MLN with its metabolite in plasma		
Reported HPTLC^12^	**-SP:** Silica gel 60F254 thin layer chromatography plates **-MP:** methylene chloride: ethyl acetate: methanol: 25% ammonia (6:3:4:1, v/v/v/v) **-RT:** 34 min per 20 samples	**Range:** **LOD:** **LOQ:**	2.75–100 1.21 3.66	simultaneous determination of Favipiravir, MLN, and ritonavir		

Lastly, we used the more updated AGREE metric to provide a more thorough, inclusive, and concise evaluation of methods' greenness, as well as an interdisciplinary rating of the proposed and published methods based on the key principles of GAC. This tool is a downloaded program that, after entering the method's 12 characters, creates a colorful pictogram with 12 portions, each colored with a different intensity of color ranging from deep green (1) to deep red (2). Furthermore, it provides a numerical value in the circular pictogram's center that is a fraction of 1; the closer the ultimate score is to 1, the greener the method is. The use of this unique approach resulted in a more useful and numerical characterization of the methods, Table 8. The greenest approach was again the published micellar HPLC–DAD method^11^ (score 0.89), followed by the reported HPLC–DAD method^9^ (score 0.85), the proposed HPLC–DAD method and the spectroscopic method^11^ (score 0.84), the HPTLC-DAD method^12^ (score 0.83), and finally the reported bioanalytical LC–MS/MS method^13^ (score 0.68). From the aforementioned results, our proposed method was found green, Table 8.

The innovative AGREE method was the sole metric that could provide a more integrative and informative rating of the analytical methods. Even though the GAPI tool is even less instructive, it supports the AGREE rank in both reported and proposed methods.

The use of three assessment tools for evaluating methods’ greenness, the Analytical Eco-Scale, GAPI, and AGREE, gives a complementary emphasis on the degree of methods’ greenness^37^.

### Assessment of whiteness of the proposed methods versus published methods

Regardless of the greenness appraisal of analytical methods, we can’t ignore that the aim of any analytical study plays a major role in choosing the best chromatographic conditions, instrumentation, and sample preparation steps. Our proposed method was superior to the other reported HPLC-DAD^9^ in that we analyzed MLN and its main DP, NHC but the reported method aimed to analyze MLN in presence of its DP only without quantifying the latter. This brought out the need for a perfect chromatographic condition to get the best resolution and peak symmetry even if a less green solvent, ACN, was needed. On the other side, the greenest micellar HPLC–DAD method^11^ was developed to determine two different drugs with different columns affinity which didn’t call for ideal separation method. This can be seen clearly in the chromatograms of this method showing obvious high tailing factors of both drugs which reflect on the relative higher LOD and LOQ than the proposed and the other reported method^9^. In addition, the reported bioanalytical HPLC–MS/MS method^13^ was classified as the least green one due to the various preparation and handling steps which were unavoidable for extracting MLN and its metabolite NHC from biological media. Moreover, using the highly energetic consumption detector, tandem mass, is the best choice for biological analysis or degradants characterization for accurate and inadequate determination of MLN.

The proposed stability indicating HPLC–DAD approach was compared to the four previously published studies that used different techniques such as HPLC–MS/MS^13^, HPTLC-DAD^12^ and HPLC-DAD^9,11^ using the RGB 12 model^20^ allowing the five methods to be compared in one excel spreadsheet. Nowak et al.^20^ released a new version of the Red Green Blue (RGB) 12 algorithm in 2021, which is divided into three groups. Each group, the red, green, and blue, comprises a color and includes characteristics that assess key components of the analytical technique. In addition to the scope of application, the red group assesses analytical efficiency in terms of validation criteria such as precision, accuracy, lowest LOD, and LOQ. The GAC principles are assigned to the green region, while productivity items such as cost and time efficiency, minimal practical requirements, and operational simplicity are assigned to the blue region. The RGB 12 model is described as an Excel file that complies with White Analytical Chemistry (WAC) guidelines. It gives a simple review of the methodologies based on the 12 WAC assumptions and evaluates sustainability in terms of whiteness assessment. A well-balanced analytical procedure is defined as "white" in the WAC approach. The proposed methodology was investigated and objectively compared to the four existing procedures that had been published. Table 9 summarizes the results of the RGB 12 model's examination of all these methods. For the validation criteria (red zone), the highest value was for the reported biological HPLC–MS/MS method (110%). The MS/MS detector was granted an extra ten merit points. This was explained in the original RGB 12 algorithm model for evaluating unique data, MLN beside its metabolite NHC, complex matrices, in addition to its lowest LOD and LOQ. In the second place was our proposed stability indicating HPLC–DAD method with 97.5% then, in the third place was the reported micellar HPLC–DAD with 95%. In this context, it is important to mention that our proposed method was of higher degree of greenness compared with the other reported method (92.5%)^9^, which comes in the fourth place, due to its wider scope of application; both separated the drug of interest from structurally similar DPs but our method was extended to kinetics degradation study and determination of the main DP, NHC. The fifth method in the red score was the HPTLC-DAD^12^ with 90% due to its ability for the determination of three drugs simultaneously which gave it a push in the red score. Finally, the spectroscopic method^11^ was the last due to its least sensitivity, linearity range 6–22 µg/mL. The scope of application in the different methods varies from the analysis of complex matrices, kinetics degradation study, separation of the drug of interest from structurally similar DPs to determination of drug mixtures.

**Table 9:** Comparison of whiteness evaluation of the proposed HPLC–DAD method together with reported methods using RGB12 model.

Method number	Method name	R (%)	G (%)	B (%)	Whiteness (%)
1	Proposed HPLC–DAD	97.5	88.8	92.5	92.9
2	Reported HPLC-DAD^9^	92.5	90.0	92.5	91.7
3	Reported micellar HPLC-DAD^11^	95.0	93.8	88.8	92.5
4	Reported biological HPLC–MS/MS^13^	110.0	82.5	85.0	92.5
5	Reported HPTLC-DAD^12^	90.0	80.0	91.9	87.3
6	Reported spectrophotometry^11^	82.5	100.0	90.4	91.0

For the green zone, discussing the environmental aspects, the spectroscopic and the micellar HPLC–DAD method showed the highest values, 100 and 93.8%, respectively due to the use of green reagents. Then, the reported HPLC–DAD method had a little bit higher score than the proposed method, 90 and 88.8, respectively due to less solvent consumption (0.5 mL/min flow rate, check Table 8). Last, the HPLC–MS/MS and HPTLC-DAD had the least greenness, 82.5% and 80%, respectively. The MS/MS detector was the highest in power consumption and the HPTLC method was the highest in the pictograms score of the solvent used and thus the least in user safety, despite being the least in the amount consumption 14 mL/ 20 spots.

The blue zone was concerned with the productivity and sustainability. HPLC–DAD methods then the HPTLC-DAD were the top in cost-effectiveness, time-efficiency, and ease-to-manipulate methods (92.5–91.9%). However, the micellar HPLC–DAD had a low score (88.8%) which was attributed to the expensively used core shell column and mobile phase. The reported HPLC–MS/MS method required special extraction technique for the assay of MLN with its metabolite in biological samples besides its high cost, thus it had the least score (85%). One of the major drawbacks of spectroscopic methods, which is reflected in its blue score, was the high sample consumption (about 3 mL). HPTLC-DAD and spectroscopic protocols employed a semi-automated and manual operation, respectively while LC–MS/MS methods used auto-injectors. As long as the published articles did not mention any transportation or special requirements for analysis, the measurements and laboratory work were presumed to be done in the same lab.

**Figure 10:**
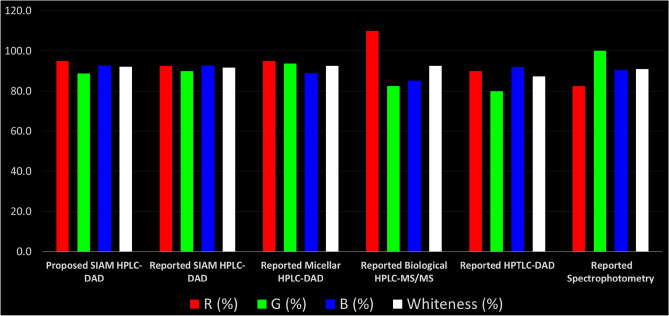
Comparison of the main assessment outcomes obtained from the RGB 12 analysis; the white line indicates 100%—a full appropriateness for planned application. The values above 100 indicate additional capabilities exceeding current requirements.

**Figure 11:**
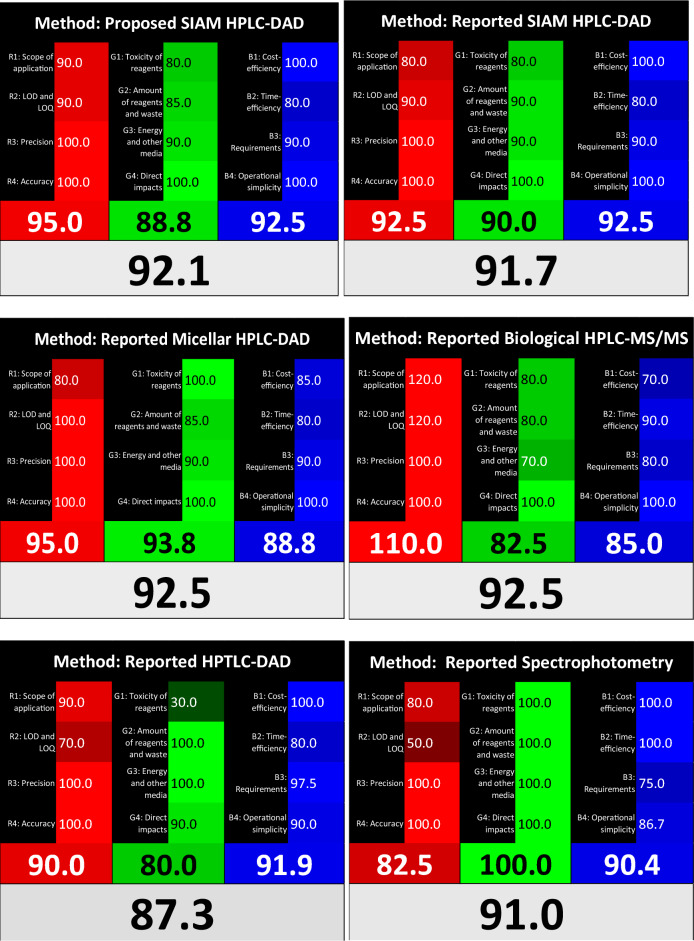
Comparison of the five model methods for the determination of MLN according to the 12 principles of WAC, performed using the RGB 12 algorithm.

Figures 10 and 11 and Table 9 illustrate the results of this extensive whiteness investigation. Our proposed method had the highest whiteness score, 92.9% compared with the other reported methods^9, 11, 13^. Moreover, the reviewed methods had high white scores (values above 87.3%) with the HPLC–MS/MS and the micellar HPLC–DAD in the top with a score of 92.5%, followed by the reported HPLC–DAD which came in the third place with a score around 91.7% followed by the spectroscopic method, 91%. The Last was the reported HPTLC-DAD with a score of 87.3%.

## Conclusion

For the first time, a rapid, reliable, and selective HPLC–DAD approach was developed to determine MLN and NHC simultaneously followed by kinetics degradation study. MLN is highly susceptible to alkali hydrolysis and to a lesser extent to acid then dry heat according to a thorough stress stability study. On the other hand, MLN was found stable under oxidative, neutral, and sunlight induced degradation conditions. Degradation rates of acid and alkali hydrolysis were also investigated, and kinetics were discovered to follow pseudo first-order. The suggested HPLC–DAD method made a significant use of DAD for peak purity assessment by the wavelength selection for the analysis. The mechanism of degradation pathways of the selected stress conditions were proposed using LC–MS-UV. The proposed DPs toxicities were assessed using ProTox-II and they were found to have negligible toxicity as the parent drug, MLN. The HPLC–DAD method's applicability as a stability-indicating approach is demonstrated by its successful analysis of MLN in marketed pharmaceutical formulations without interference from typical capsule excipients or probable degradation products. Finally, a thorough comparison with the reported methods for MLN analysis with greenness and whiteness appraisal was done and it was found that our proposed method for MLN determination was of superior whiteness.

## Materials and methods

### Materials and reagents

MLN and N4-hydroxycytidine (NHC) were purchased from Sigma-Aldrich Chemie GmbH, Swizterland Germany with purity 98%. Molnupiravir-Eva Pharma® tablets were purchased from the Egyptian market. Analytical grade hydrochloric acid (HCl), sodium hydroxide (NaOH), and hydrogen peroxide (H_2_O_2_) 30% were all purchased from El-Nasr Chemical Industry Company, Egypt. Both acetonitrile (ACN) and methanol (MeOH) were HPLC-grade and were purchased from Baker (Ireland). The water used was deionized and it was filtered before use. Syringe filters, 0.2 µm 25 mm PTFE, were purchased from Ultrapure, China.

### Apparatus

HPLC–DAD system consisted of Agilent 1200 series (auto-injector, quaternary pump, vacuum degasser and diode array multiple wavelength detector G1315 C/D and G1365 C/D) connected to a computer loaded with Agilent ChemStation Software (Agilent Technologies, Santa Clara, CA, USA). The chromatographic separation was performed on an Agilent HC-C18 analytical column (150 × 4.6 mm, 5 µm).

LC–MS-UV system utilized was Shimadzu® UFLC series (Shimadzu Corporation, Kyoto, Japan) coupled with two detectors: UV detector SPD-20A and MS detector LCMS-2020 with single quadrupole system and two ion sources ESI or APCI. Mass range of m/z 10 to 2000 and a scan rate of up to 15,000 amu/sec were used. The stationary phase was a Shim-pack XR-ODS II® C18 analytical column (100 × 3 mm, 2 µm) (Shimadzu, Japan).

### Chromatographic conditions

#### HPLC–DAD

Separation was performed on an Agilent HC-C18 analytical column (150 × 4.6 mm, 5 µm). The mobile phase system consisted of solvent A (deionized water) and solvent B (ACN) in an isocratic elution 75:25, v/v, respectively. Water was filtered by passing through 0.45 µm millipore membrane filter and a temperature of 25 ºC was used. The detection wavelength using DAD was set at 236 nm. The total run time was 5 min with 1 mL/min flow rate and 20-µL injection volume.

#### LC–MS-UV

Shim-pack XR-ODS II ® C18 analytical column (100 × 3 mm, 2 µm) was used. The mobile phase system consisted of solvent A (deionized water) and solvent B (ACN) in an isocratic elution 80:20, v/v, respectively with detection operated with electrospray ionization (ESI) interface adjusted on scan mode: selected ion monitoring (SIM) with positive and negative ionization polarity. Water was filtered by passing through 0.45 µm millipore membrane filter and a temperature of 25 ºC was used. The total run time was 5 min with 0.4 mL/min flow rate and 10-µL injection volume.

### Preparation of stock and standard solutions and construction of calibration graphs

Stock solutions of MLN and NHC 1000 µg/mL were prepared in HPLC-grade methanol. Solutions were stored at 0 °C in freezer. Dilution of the stock solutions using deionized water was performed to get working standard solutions within the concentration range 0.1–100 µg/mL. The injection of each concentration into the HPLC–DAD system under the optimized corresponding conditions was made in triplicate using 20 µL volume of injection. Peak areas of MLN and NHC recorded at their R_t_ of 3.7 ± 0.05 and 2.9 ± 0.06 min, respectively, were related to the corresponding concentrations to get the regression equation and the calibration graph.

### Analysis of tablets

Five Molnupiravir-Eva Pharma® capsules were opened and the powder was weighed (each pill was labelled to contain 200 mg MLN). A quantity equals to one capsule's average weight was properly transferred into a 100-mL volumetric flask, dissolved with 60 mL methanol, and sonicated for about 10 min. The mixture was then completed to volume with methanol and filtered using 0.22 µm particle size membrane filters. Appropriate dilution was then made to get a concentration equivalent to 50 µg/mL. In addition, different known concentrations of pure MLN standard were spiked to different aliquots of the prepared tablet solution (50 µg/mL) (standard addition method) and processed as stated previously.

### Forced degradation and stability-indicating study

Forced degradation studies were done using separate volumes of 1 mL of MLN stock solution (1000 µg/mL). After being exposed to different degradation conditions, the solutions were then completed to final volumes in 10-mL volumetric flasks using deionized water to reach a final concentration of 100 µg/mL for each degradation case according to the following conditions:

### Acidic and alkaline hydrolysis

MLN solutions were treated with 1 mL volumes of 2 M HCl. Mixtures were then kept in a thermostatic water bath at 60 ˚C for 30 min (for acidic hydrolysis). For alkaline hydrolysis, MLN solutions were treated with 1 mL volumes of 0.01 M NaOH. Solutions were left at room temperature for 2.5 h. After the specified time interval, for both acidic and alkaline hydrolysis, the flasks were cooled, if needed, then neutralized to pH 7. Final volumes were completed with deionized water to reach a specified final concentration of 100 µg/mL in 10-mL volumetric flasks.

### Neutral hydrolysis

Separate volumes of 1 mL of freshly filtered deionized water were added to MLN solutions. Mixtures were heated in a thermostatic water bath at 80 ˚C for 30 min, cooled and completed to volume as illustrated above.

### Oxidative degradation

MLN solutions were treated with 1 mL of hydrogen peroxide (30% v/v), heated in a thermostatic water bath at 60 ˚C for 2 h, cooled then completed to volume as illustrated above.

### Dry heat degradation

For dry heat degradation, 10 mg portions of MLN dry powder were placed in a porcelain dish in a temperature-controlled oven at 100˚C for 2 h. The content of the porcelain dish was then quantitatively transferred with HPLC-grade methanol into a 10-mL volumetric flask and completed to volume using the same organic solvent. Then, an aliquot of this methanolic stock solution was diluted to obtain a final concentration of 100 µg/mL as illustrated above.

### Photolytic degradation

Photo- stability study was performed by exposing 10-mL volumetric flask containing MLN stock solution (1mL) to sunlight during daytime for 4 h. After the specified time, the volume was completed as illustrated above.

### Kinetics investigation

To investigate degradation kinetics, 1mL volumes of MLN stock solution (1000 μg/mL) were subjected to acid (2 M HCl) and alkali (0.01 M NaOH) stress conditions, at room temperature for different time intervals. Each time, samples were neutralized, diluted to 10 mL volumes with deionized water, and analyzed sequentially by the proposed HPLC–DAD method to monitor the decrease in MLN concentration with time utilizing its regression equation.

### LC–MS-UV study on the intact drug and its degradation products

Structural elucidation of MLN degradation products (DPs) under acidic, alkaline, and dry heat stress conditions was accomplished using LC–MS-UV. DPs were generated under acidic (2 M HCl, 60 °C, 1 h), alkaline conditions (0.01 M NaOH, 60 °C, 1 h), and dry heat (100 °C, 2 h). Degraded solutions were then neutralized, if needed followed by dilution, as mentioned above. At first, a concentration of 100 μg/mL of MLN was injected into the LC–MS-UV system to allocate the drug peak and its corresponding m/z using the optimized parameters. Secondly, acidic, alkaline, and dry heat degradation solutions were introduced into LC–MS–UV to allocate DPs peaks. The peaks of MLN and its possible DPs were monitored in positive as well as negative scan mode using electrospray ionization (ESI) with mass range from 100 to 1000 m/z.

### In-silico toxicity studies

The toxicity of MLN and its major DPs (DP1, NHC and DP2, both formed under acid, alkaline, and dry heat stress conditions), was predicted in silico using the Pro Tox- II web server program. For the validation of ProTox-II acute toxicity model, leave-one-out cross-validation is used. ProTox-prediction II's parameters have been improved to boost toxicity class and LD50 prediction hit rates. Toxic doses are frequently expressed as LD50 values in milligrams per kilogram of body weight. The median lethal dose (LD50) is the dose at which half of test subjects die after being exposed to a substance^18, 19^.

### Greenness and whiteness of the method

In this study, different techniques have been applied for greenness assessment of the proposed analytical method in comparison with the published reports for MLN determination. These techniques include the Analytical Eco-Scale tool, the green analytical procedure index (GAPI), and the Analytical greenness metric (AGREE). Mainly, it is better to combine several greenness evaluations tools to generate a more thorough comparison and objective ranking of various analytical methods in agreement with their eco-friendliness, and this was appointed in many recently published analytical reports.

As an extension of Green Analytical Chemistry (GAC), the theory of White Analytical Chemistry (WAC) was developed. As an alternative to the well-known 12 GAC principles, Nowak et al.^20^ suggested the 12 WAC principles. WAC considers other significant variables determining the method's quality, including as analytical (red) and practical (blue) elements, in addition to green features (blue).

## Statistical analysis

Data analysis tool/ Microsoft Excel software packaged with Microsoft Excel 360 was used for regression analysis and calculation of validation parameters. Pro Tox- II web server program was used for evaluating the in-silico toxicity. AGREE downloadable program, supported with Excel software, was used.

### Supplementary Information

Supplementary Information.

## Data Availability

The datasets generated during the current study are available from the corresponding author on reasonable request.
