# Supplementary material for: White targeted chromatographic screening method of Molnupiravir and its metabolite with degradation kinetics characterization and in-silico toxicity
Source: Sci Rep. 2023 Oct 20;13:17919. doi: 10.1038/s41598-023-44756-6 (PMC10589288; doi:10.1038/s41598-023-44756-6)
Supplement: Supplementary file 1 — Supplementary Information. [file 41598_2023_44756_MOESM1_ESM.docx]

a)


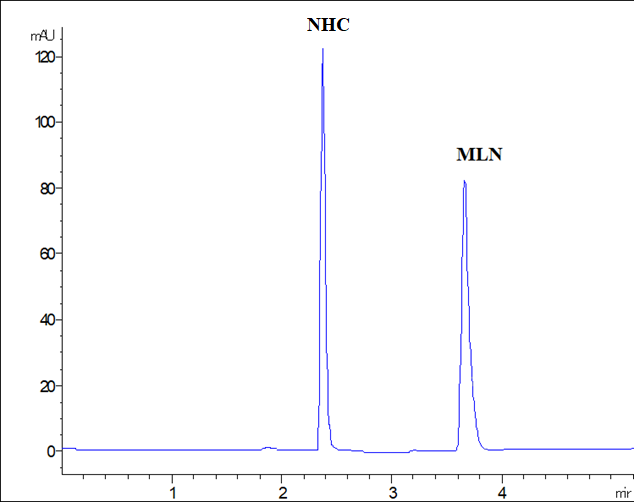


b)


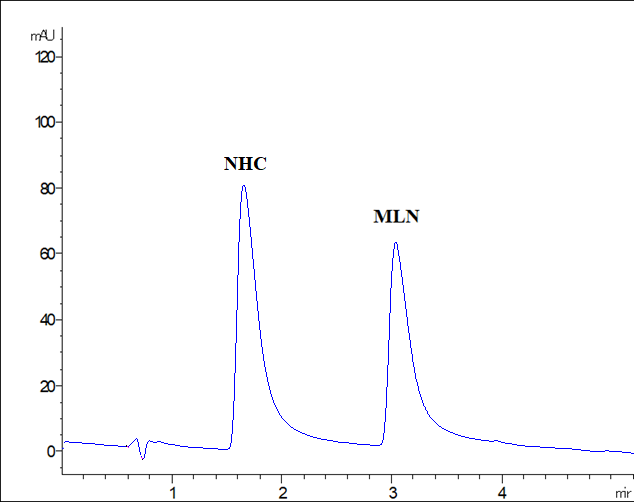


**Supplementary Figure 1.** **HPLC DAD chromatogram of 10 µg/mL MLN and NHC using methanol, a), compared with acetonitrile, b) as organic modifiers.**

**a)**


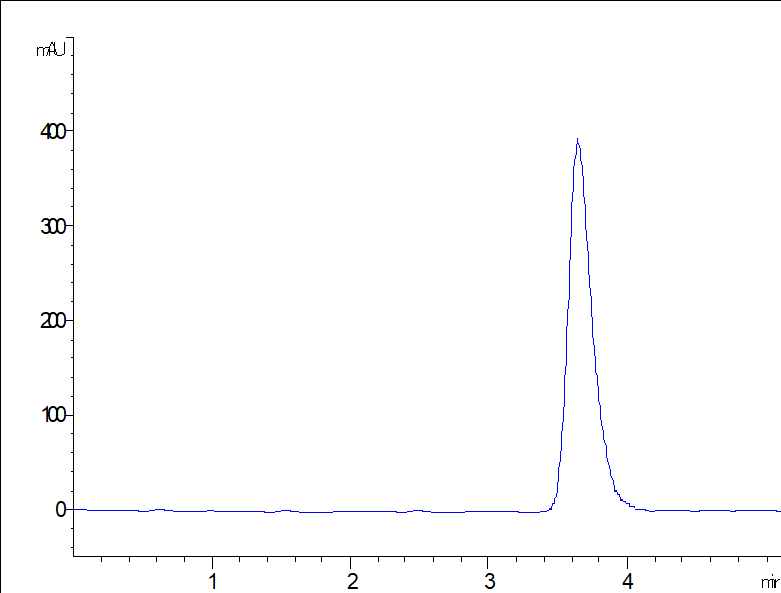


b)


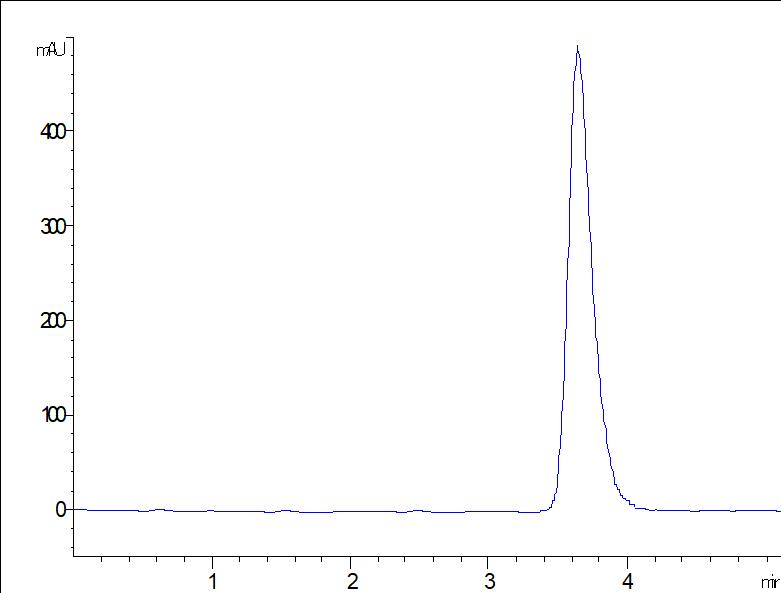


**Supplementary Figure 2. HPLC DAD chromatogram of a prepared solution of MLN capsules, 50 µg/mL MLN, a), and after spiking with a standard MLN (10 µg/mL), b).**
